# Reusable Tourniquets as Potential Transmitters of Infection: A Microbiological Analysis

**DOI:** 10.3390/microorganisms13010152

**Published:** 2025-01-13

**Authors:** Julia Szymczyk, Monika Kurpas, Bartosz Krasiński, Katarzyna Zorena, Wioletta Mędrzycka-Dąbrowska

**Affiliations:** 1 Division of Anaesthesiology Nursing & Intensive Care, Faculty of Health Sciences, Medical University of Gdansk, 80-211 Gdansk, Poland; juliaszymczak@gumed.edu.pl; 2 Division of Immunobiology and Environmental Microbiology, Faculty of Health Sciences, Medical University of Gdansk, 80-211 Gdansk, Poland; monika.kurpas@gumed.edu.pl (M.K.); katarzyna.zorena@gumed.edu.pl (K.Z.); 3 Emergency Department, Health Care Entity Copernicus Sp. z o. o., 80-462 Gdansk, Poland; wiomed@wp.pl

**Keywords:** healthcare-associated infections (HAIs), reusable tourniquets, bacterial contamination, medical equipment disinfection, device-associated healthcare infections (DA-HCIs), hygiene in healthcare, catheter-related bloodstream infections (CRBSIs)

## Abstract

Introduction: Healthcare-associated infections (HAIs) pose a significant global challenge, resulting in prolonged hospital stays, higher healthcare costs, and increased morbidity and mortality rates. Reusable medical equipment, such as tourniquets, represents a potential vector for infection transmission. Despite frequent use and close contact with patients’ skin, infection control protocols often overlook these devices. This study examines microbial contamination on the surface of reusable tourniquets in both emergency department and operating theatre settings. Methods: A cross-sectional study was conducted between March and September 2024 in Gdansk, Poland. Samples from tourniquets used in the emergency department and the operating theatre were collected after an indefinite period, 14 days, and 28 days. Bacterial contamination on the surfaces of the tourniquets was measured using Columbia agar blood medium and expressed as colony-forming units (CFUs) per cm^2^. Results: Significant bacterial loads were detected on reusable tourniquets, with contamination levels varying by location and duration of use. The average number of CFU/cm^2^ across all stages of this study was 545 CFU/cm^2^ for the emergency department and 101 CFU/cm^2^ for the operating theatre. Tourniquets used in the emergency department exhibited higher bacterial counts compared to those from the operating theatre, which showed a greater diversity of bacterial species. These findings underscore the need to revise infection control protocols for reusable tourniquets. Conclusion: This study provides critical data that may influence future policy changes aimed at reducing the risk of HAIs through the improved management of reusable medical devices.

## 1. Introduction

Healthcare-associated infections (HAIs) represent a major public health concern worldwide, contributing to prolonged hospital stays, increased healthcare costs, and higher morbidity and mortality rates. It is estimated that millions of patients worldwide contract infections during their hospital stay, with many cases directly linked to contaminated medical equipment and inadequate hygiene practices. The World Health Organization (WHO) has identified nosocomial infections as a critical issue, urging healthcare providers to adopt stricter infection control measures and improve sterilization practices for medical devices [1]. In this context, reusable medical devices, such as stethoscopes, blood pressure cuffs, and tourniquets, have attracted increasing attention due to their potential to transmit multidrug-resistant pathogens [2,3,4,5,6].

Among reusable devices, tourniquets are particularly prevalent in healthcare settings, used for intravenous (IV) cannulation, diagnostic test sample collection, and drug administration. Tourniquets, typically elastic bands, are applied to visualize veins before accessing the vascular system. For this purpose, the band is placed on the patient’s limb, 10–15 cm above the intended insertion site. Despite their frequent use and direct contact with the patient’s skin, reusable bands are often overlooked in infection control protocols [7]. Tourniquets are used across a wide range of patients, regardless of infection risk, and are seldom disinfected between uses, despite WHO and PTPI guidelines recommending that tourniquets and other non-critical items be either disposable or cleaned between uses [8,9].

**Figure 1:**
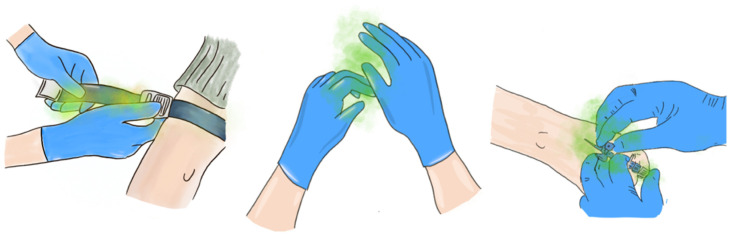
Illustration of the microbial transmission pathway from a reusable tourniquet to a peripheral cannula.

Unlike surgical instruments, which are sterilized between patients, tourniquets are rarely, if ever cleaned, leading to the accumulation of contaminants over time. This practice poses a significant risk, especially in environments where immunocompromised patients are treated, such as intensive care units (ICUs) and oncology wards [10]. The transfer of microorganisms via reusable tourniquets may increase the risk of infection, leading to inflammation at the insertion site and exposing immunocompromised patients (Figure 1). Research by Perreira et al. has demonstrated that using disposable tourniquets can reduce complications related to peripheral intravenous cannula (PIVC) insertion [11].

Previous studies have identified various bacterial species on medical devices, including *methicillin-resistant Staphylococcus aureus* (MRSA), *Escherichia coli*, and *Pseudomonas aeruginosa*—organisms known for their virulence and antibiotic resistance. However, most existing studies have focused on larger medical instruments and equipment, such as surgical and hospital beds, with limited attention given to smaller reusable devices such as tourniquets. Thompson et al. demonstrated high levels of bacterial contamination of reusable blood pressure cuffs, suggesting that frequency of use and proximity to patients’ skin may be key factors in spreading bacteria on surfaces. This raises critical questions about whether reusable tourniquets with similar use patterns may also serve as reservoirs for bacterial transmission [12]. Tourniquets, often reused for many patients without proper cleaning, can contribute to the spread of these resistant organisms, undermining infection control efforts [13].

The purpose of this study was to compare microbial contamination on the surface of reusable tourniquets in emergency departments and operating theatres. The side aims were to investigate whether the type of office and the duration of use of the reusable tourniquet has an impact on the number of colony-forming units per cm^2^.

## 2. Material and Methods

### 2.1. Study Design

This research was conducted as a cross-sectional study.

### 2.2. Settings

This study was conducted between March and September 2024 in one of the hospitals in Gdansk, Poland. The researchers followed the relevant legal regulations and the bioethics principles of the Declaration of Helsinki, paying due attention to the Strengthening the Reporting of Observational Studies in Epidemiology (STROBE) criteria [14]. This study received approval from the Independent Bioethics Committee for Research of the Medical University of Gdansk (approval number: KB/45/2024, 29 February 2024). This project was registered on ClinicalTrials.gov (NCT06566495).

### 2.3. Samples

**Figure 2:**
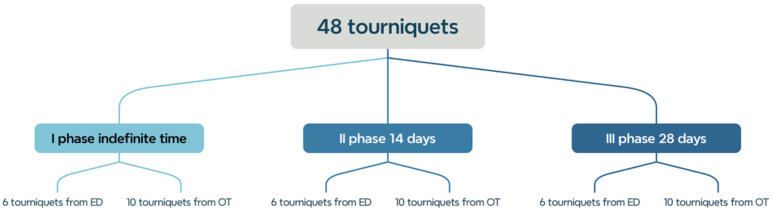
Diagram showing the stages of the study and the planned number of tourniquets.

It was assumed that a total of 48 tourniquets would be collected in all three stages (Figure 2). The main parameters in this study were the length of time the tourniquets were used by medical staff and the characteristics of the ward. This study used reusable tourniquets made of flexible material purchased from the same manufacturer and delivered in their original packaging to specific hospital units for regular use during vascular access fabrication in the anesthesiology and emergency departments. The study groups of tourniquets were defined as follows: tourniquets after an indefinite period of use (indefinite group), tourniquets after 14 days of use, and tourniquets after 28 days of use. Indefinite time refers to the tourniquet group we encountered in the hospital that did not have a specific time frame imposed as in the other groups of 14 days and 28 days. The group with an indefinite time served as the baseline for comparison with the other two groups. The time of use was chosen to avoid overextending the time of use and to determine whether doubling the time would be associated with a linear temporal accumulation of bacteria or whether the accumulation and generic composition of the bacteria was a stochastic process.

### 2.4. Characteristics of Sampling Locations

The emergency ward comprises child and adult observation rooms, a pediatric office, and a resuscitation area. The operating theater performs procedures in general surgery, orthopedics, urology, gynecology, neurosurgery, and pediatrics. Another variable was how multiple tourniquets were handled by staff in the various departments. These ways included, but were not limited to, the frequency with which tourniquet procedures were performed, whether they were decontaminated after use, the type of disinfectant used for decontamination, and how the tourniquets were stored and transported to patients. Such variables may or may not have influenced the bacteriome’s qualitative and/or quantitative composition. Still, the non-linearity of these variables, related to the circumstances of tourniquet use and the individual approach of the staff to their use, are factors that are not subject to strict regulations and cannot be recorded under everyday conditions. In this study, we emphasized the specificity of the wards. Operating theatres are some of the most closely monitored rooms in terms of microbiological cleanliness and have limited access for staff and patients. The second choice was the emergency wards, which are characterized by the high dynamics of handling patients with various conditions, including mechanical injuries often associated with blood loss and differing levels of patient hygiene.

### 2.5. Collection of Material

Reusable tourniquets from the anesthesiology and emergency departments were collected in disposable, sterile bags. All the polyamide tourniquets tested were from the same manufacturer, which states in the product specification that the bandage should be sterilized at 80 degrees C. All tourniquets were labelled and assigned to different rooms within the wards examined. Tourniquets that were collected after the time of use were immediately packaged in sterile bags (Interscience BAGPAGE, Saint Nom la Bretêche, France) and transported at room temperature to the Department of Immunobiology and Environmental Microbiology at the Medical University of Gdansk for microbiological screening 3 h after collection of the tourniquets from the hospital wards. After each of the in-hospital stages of the study, the tourniquets were collected and replaced with new ones.

### 2.6. Enumeration of Microorganisms on Tourniquet Components

Biological material from the reusable tourniquet was analyzed separately for the plastic clasp and the fabric band. The study utilized only solid media for microbial culturing. Columbia agar plates supplemented with 5% sheep blood were used as the solid medium, providing a nutrient-rich environment that supports the growth of a wide range of microorganisms. Additionally, it allows for the observation of hemolysis, which can provide insights into certain microbial activities. Colony-forming units (CFUs) were counted on the agar plates after incubation, based on colony formation.

#### 2.6.1. Plastic Claps

##### Study on Columbia Agar (Total Microbial Count)

Under laboratory conditions, the plastic parts of the tourniquets were cut off and placed in sterile dishes. Using sterile swabs soaked in 0.9% NaCl, samples were collected from the plastic surface and placed in a tube containing 3 mL of 0.9% NaCl. The tube was then shaken for 30 s using a vortex mixer (IKA-Werke, Staufen, Germany).

The resulting bacterial suspension was seeded onto Columbia agar supplemented with 5% sheep blood (GRASO Biotech, Owidz, Poland) in two forms: a concentrated suspension (200 µL) and a ten-fold diluted suspension (200 µL). The inoculated Columbia agar plates were incubated at 37 °C for 24 h under aerobic conditions. After incubation, the colonies were counted, and the results were expressed as colony-forming units (CFUs) for the entire surface of the clasp.

#### 2.6.2. Fabric Band

##### Study on Columbia Agar (Total Microbial Count)

**Figure 3:**
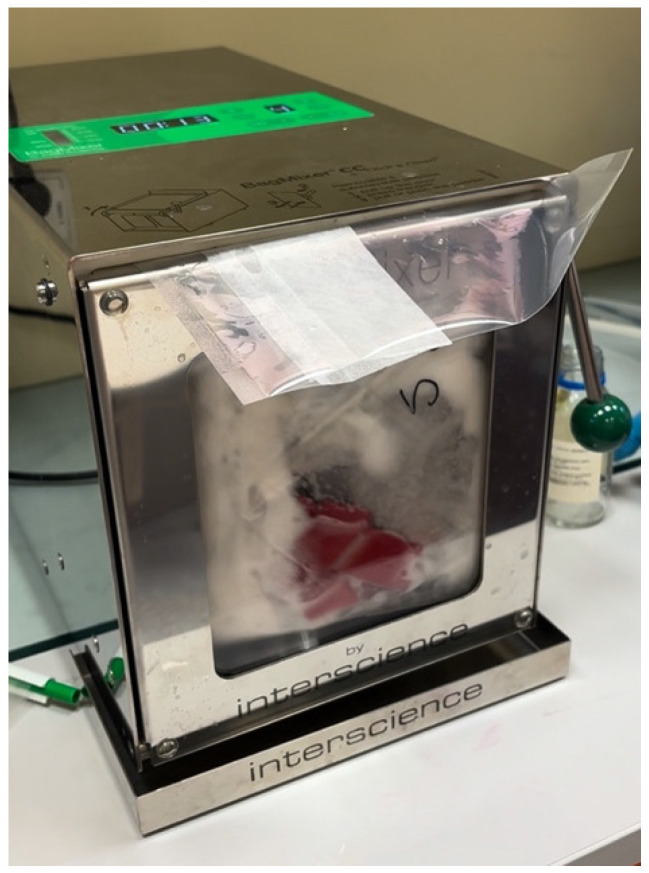
Homogenization process of the fabric part of the tourniquet.

**Figure 4:**
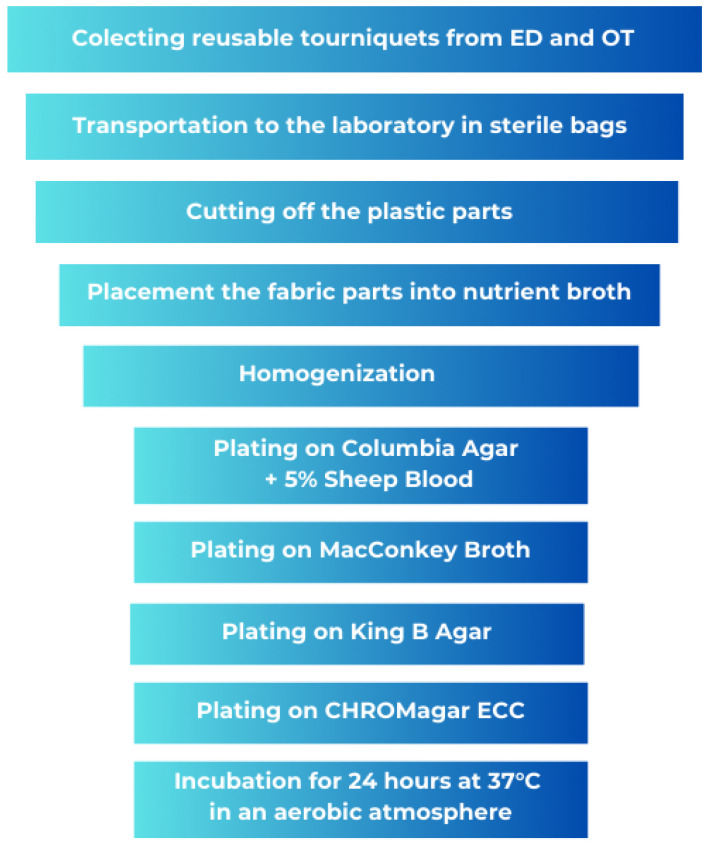
Stages of material collection. ER, Emergency Department; OT, Operating Theatre.

The material part of the tourniquet was placed in a sterile homogenization bag with 100 mL of nutrient broth. The contents of the bag were then homogenized in a BagMixer 400 CC stomacher (Interscience, Saint-Nom-la-Bretèche, France) for two minutes to detach the microorganisms from the porous surface of the material (Figure 3). The resulting homogenate from each sample was transferred in a concentrated form, and ten-fold and hundred-fold dilutions were prepared using 9 mL of nutrient broth for each dilution. Each dilution, as well as the concentrated homogenate, was then plated once onto the surface of the Columbia agar plates supplemented with 5% sheep blood. The inoculated Columbia agar plates were incubated at 37 °C for 24 h under aerobic conditions. After incubation, the colonies were counted, and the results were expressed as colony-forming units (CFUs) per cm^2^ of fabric band. (Figure 4). Outcome measures, e.g., bacterial growth, were assessed by counting the number of colonies on the Columbia agar plates. After incubation, the bacterial colonies were counted to determine the number of microorganisms on the surface of the tourniquet (expressed as CFU/cm^2^) or on the whole clamps in the case of plastic parts. Additionally, the optical density (OD) of the concentrated homogenate was measured using a spectrophotometer to evaluate the contamination level of the tourniquets.

##### Study on MacConkey Agar and King B Agar Plates (Gram-Negative Bacteria and Pseudomonas Count)

In addition, 200 µL of the concentrated homogenate, without any dilution, was plated onto MacConkey agar and King B agar plates. These media were selected to evaluate the diversity of microorganisms present in the samples.

MacConkey agar allows for the growth of Gram-negative bacteria while inhibiting the growth of most Gram-positive bacteria. It is especially useful for isolating and differentiating enteric bacteria based on their ability to ferment lactose.

King B agar was chosen because it is selective for *Pseudomonas* species, allowing for the growth of this genus while inhibiting the growth of other microorganisms.

All plates were incubated for 24 h at 37 °C in an aerobic atmosphere to allow for microbial growth and colony formation. These media helped in identifying the presence and diversity of microbial contamination on the samples, as well as enabling the differentiation of specific bacterial groups.

### 2.7. Statistical Methods

Data were entered and analyzed using Statistica software version 13.3 (TIBCO Software Inc., Krakow, Poland). Descriptive data were presented as frequencies, means, and percentages. Values of *p* < 0.05 were considered statistically significant. Given that the data from the emergency room and the operating room did not meet the assumption of normal distribution and given the small sample sizes (<30), the Mann–Whitney U test was used.

## 3. Results

A total of 48 samples taken from reusable bands contained bacterial colonies. Due to the assumptions of the experiment and the time frame, five tourniquets were not included in the analysis. Six reusable tourniquets were collected in each of the three stages of the study in the emergency department (eighteen in total). In the operating room, 30 tourniquets were collected in all (10 for each stage of the study).

### 3.1. Microbial Contamination

#### 3.1.1. Abundance on Columbia Agar

Microorganisms were detected in each stage, confirming their widespread occurrence in the samples analyzed. The data is shown in Table 1.

There is a statistically significant difference (*p* = 0.00001) between the emergency department and the operating room (Table 1) regarding the number of CFU/cm^2^ per tourniquet.

There is a statistically significant difference (*p* = 0.03) between 14 days and 28 days of tourniquet use regarding the number of CFUs at the study sites. No significant statistical difference was observed between 14 and 28 days of tourniquet use in the number of CFU/cm^2^ in the emergency department. The average number of CFU/cm^2^ across all stages of this study for the emergency department was 545 CFU/cm^2^ and for the operating theatre was 101 CFU/cm^2^. In comparison, in the human skin microbiome, the average number of microorganisms isolated using traditional culture methods from the skin surface ranges from 10^4^ to 10^5^ CFU/cm^2^ [15,16].

**Figure 5:**
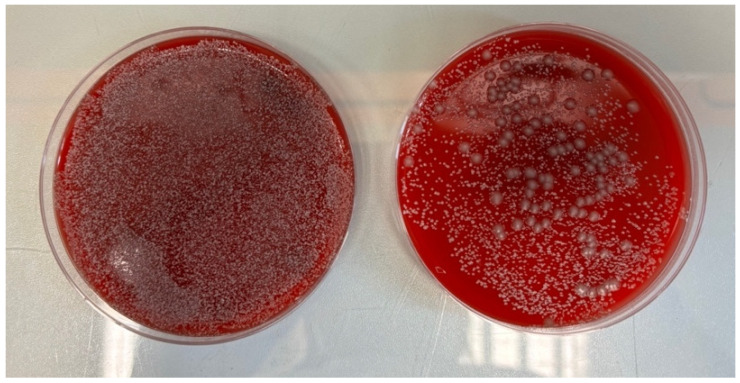
Photograph showing material obtained from tourniquets in the emergency department’s pediatric area, seeded on Columbia agar 5% medium: concentrated state on the left and 10-fold dilution on the right.

The tourniquets colonized to the highest degree were those from the emergency department, specifically the pediatric area (Figure 5) and the adult observation area.

**Figure 6:**
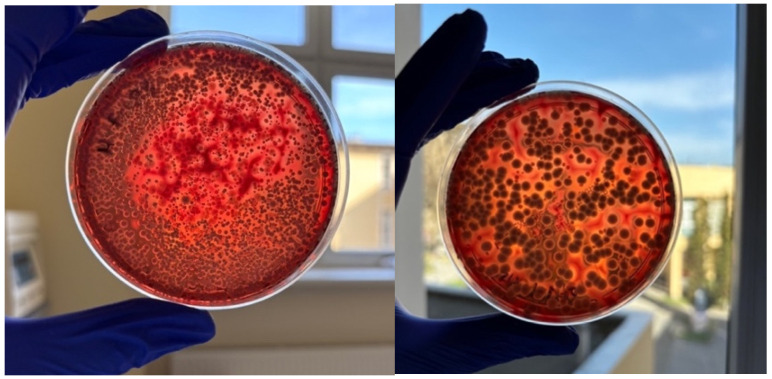
Photograph showing material obtained from tourniquets in the operating theatre’s pediatric room after 28 days, seeded on Columbia agar 5% medium: concentrated state on the left and 10-fold dilution on the right, with an uncountable number of hemolytic bacteria.

After 28 days, exceptionally high numbers of CFUs of hemolytic bacteria were observed on cultures from the operating theatre tourniquets. Figure 6 shows the media with a 10-fold diluted homogenate and a concentrated homogenate. An uncountable number of colonies of hemolytic bacteria can be seen on the agar plate.

#### 3.1.2. Abundance on Qualitative Media

For the MCC (MacConkey agar) substrate, the mean microbial count ($\bar{x}$) in the emergency department was 517 CFU/cm^2^, and in the operating theatre, it was 29 CFU/cm^2^. For the King B substrate, the mean microbial count ($\bar{x}$) in the emergency department was 517 CFU/cm^2^, and in the operating theatre, it was 207 CFU/cm^2^ (Table 2). There was no statistically significant difference in microbial count (CFU/cm^2^) between the 14-day and 28-day intervals.

### 3.2. Blood Stains

A total of 21 of the 48 tourniquets examined (44%) had visible blood stains (Table 3).

## 4. Discussion

### 4.1. Key Results

Our study, which examined the presence of microorganisms on reusable blood collection tourniquets, highlights significant safety and hygiene concerns in medical practice. Tourniquets, essential tools for blood collection, can become potential vectors for infection transmission.

#### 4.1.1. Significance of Findings

Our study revealed the presence of various microorganisms, including pathogenic bacteria, on the surface of reusable tourniquets. This finding aligns with previous research suggesting the risk of infection transmission through reusable medical equipment [17]. The dynamic environment of emergency departments (EDs), characterized by rapid patient turnover and inconsistent hygiene practices, likely contributes to higher contamination levels. Natarajan et al. emphasized the lack of established cleaning protocols for reusable tourniquets [17]. A study conducted in a Greek emergency department found high bacterial contamination on medical devices, such as stethoscopes and cardiac monitors, with 99% of devices testing positive for bacteria, primarily coagulase-negative staphylococci [18]. Similarly, a Polish emergency department study found higher bacterial presence compared to other work environments, underscoring the risk of pathogen exposure in such settings [19]. The long-term use of tourniquets, with bacteria persisting even after 14 and 28 days, raises concerns about the effectiveness of current disinfection protocols.

#### 4.1.2. Microbial Growth Analysis

Our study also examined bacterial growth on different media. On MacConkey medium, which is used to culture Gram-negative bacteria, from the Enterobacteriaceae family selectively, the average colony-forming units (CFUs) per cm^2^ were 517 in the emergency department and 29 in the operating theatre. The Enterobacteriaceae family includes various pathogens, such as *Escherichia*, *Salmonella*, *Shigella*, *Klebsiella*, *Enterobacter*, *Serratia*, *Proteus*, *Yersinia*, and *Citrobacter*, which can cause urinary tract infections (UTIs), gastrointestinal infections, respiratory infections, tissue infections, and sepsis.

On King B agar plates, used to isolate and differentiate fluorescent *Pseudomonas* species, including *Pseudomonas aeruginosa*, the average bacterial count was 517 CFU/cm^2^ in the emergency department and 207 CFU/cm^2^ in the operating room.

The third medium, ECC (*E. coli Coliforms Chromogenic*) agar, used for differentiating *Escherichia coli* and other coliforms, did not yield significant growth.

#### 4.1.3. Site and Timing of Sample Collection

We selected a tertiary referral hospital for this study due to its advanced medical services. Despite the hospital’s high standard of care, it lacks a protocol for cleaning reusable equipment. We compared tourniquets from two distinct environments: the emergency department, with its high patient volume and variable conditions, and the operating theatre, which, although cleaner, has more controlled conditions and fewer patients. This comparison underscores the stark contrast in contamination levels between these two settings. The ED is designed for rapid triage, assessment, stabilization, and treatment of patients with diverse and often urgent medical conditions. It operates in a fast-paced, noisy, and chaotic environment, with a high turnover of patients and medical staff. The focus is on quick diagnosis and stabilization, with procedures tailored to swiftly address the most critical needs.

In contrast, the operating theatre is designed for surgical procedures, where sterility and precision are paramount. The environment is controlled to minimize infection risk, and the work involves carefully planned and organized operations. Though emergencies may arise, the operating theatre’s procedures adhere strictly to surgical and aseptic protocols. Our study confirms the microbiological differences between these two areas in terms of contamination.

In terms of timing, we based our study on pre-survey results and the viability of microorganisms over time. The group with an indefinite use duration served as a baseline for the 14- and 28-day groups. Previous studies have not investigated temporal variation in microbial counts in similar settings [6]. The average number of CFU/cm^2^ across all stages of the study for the emergency department was 545 CFU/cm^2^ and 101 CFU/cm^2^ for the operating theatre. In comparison, in the human skin microbiome, the average number of microorganisms isolated using traditional culture methods from the skin surface ranges from 10^4^ to 10^5^ CFU/cm^2^ [15,16].

#### 4.1.4. Septic Infections

Catheter-related bloodstream infections (CRBSIs) are a leading cause of sepsis, associated with increased morbidity, mortality, and significant medical costs ($32,000 to $69,332) [10,11,12,13,14]. The microorganisms most commonly responsible for CRBSIs include *Acinetobacter* spp., *Klebsiella pneumoniae*, coagulase-negative staphylococci, and *Candida albicans* [20].

#### 4.1.5. Blood Stains

Blood stains were identified on the surface of 44% of the tourniquets tested. It would be valuable to investigate whether blood-borne viral infections, such as HBV, HCV, and HIV, could be related to blood stains on the tourniquets.

#### 4.1.6. Material Collection Method

Earlier studies often used broth mixture methods [21,22], but microbial swabbing has become more common for material collection [23,24,25]. Other studies have imprinted the surface of tourniquets on microbiological media [26,27,28]. We employed a method involving mechanical shaking to remove the microorganisms from the porous structure of the tourniquets, which allowed for a more accurate microbiological analysis. Although swabs collect smaller amounts of material, they may not be sufficient for certain tests, especially when microorganism concentrations are low. Additionally, swabs carry a risk of contamination during collection or transport if sterility is not maintained. In contrast, homogenization ensures a more representative and accurate sample by allowing a larger volume of material to be collected, which is particularly beneficial for tests requiring larger sample sizes.

#### 4.1.7. Impact on Medical Practices

The presence of microorganisms on tourniquets highlights the need for stricter disinfection and sterilization protocols in healthcare facilities, or the use of disposable tourniquets. Leitch et al. noted that disposable tourniquets are unpopular due to the difficulty of insertion and discomfort caused by the rubber material [24]. Golder et al. emphasized the importance of following protocols to prevent cross-contamination, not only regarding hand hygiene but also with reusable equipment [28]. Our findings suggest that standard cleaning methods may not be adequate, and more advanced techniques, such as chemical disinfection or steam sterilization, might be necessary to effectively eliminate pathogens. However, the consistency with which these methods are implemented must also be considered. The reusable tourniquets available in Poland lack clearly defined cleaning guidelines in their product specifications. Some sources recommend sterilization at 80 °C, limiting autoclaving options to remove spores. To address this, a higher temperature of 121 °C should be used, though this could distort the tourniquets. According to hospitals in Pomerania, no internal cleaning procedures exist for reusable tourniquets. This lack of standardization and manufacturer guidelines points to the need for developing and standardizing hospital protocols.

Additionally, medical staff should be educated on the importance of hand hygiene and regular equipment disinfection to minimize infection risks. Mehmood et al. observed a lack of awareness among medical staff regarding the spread of pathogens via reusable tourniquets and highlighted insufficient staff involvement in the disinfection process [25].

#### 4.1.8. Proposals for Further Research

Future studies should investigate the correlation between tourniquet material types and microorganism adhesion. Studies on the effects of various materials on microbial survival and multiplication would be valuable. Additionally, the impact of incorporating metal nanoparticles into reusable equipment could be examined, as antimicrobial coatings could improve infection control measures and extend the lifespan of tourniquets, potentially reducing medical waste.

Life cycle assessment (LCA) studies should also be conducted to comprehensively evaluate the environmental impact of disposable versus reusable tourniquets. This would help to determine which option has less ecological impact, considering factors such as distribution, plastic use, and disposal.

### 4.2. Limitations

Our study was limited by a small sample size (*n* = 48) and a narrow range of microorganisms analyzed. Future research should include larger samples and examine a broader spectrum of microorganisms to obtain more comprehensive data on the risks associated with reusable tourniquets. We did not monitor how staff handled the tourniquets, including disinfectant use, during this study.

### 4.3. Conclusions

In summary, our study provides evidence of significant microbial contamination on reusable blood collection tourniquets, with differences in species and abundance across hospital settings. These findings underscore the infection risks associated with reusable tourniquets, reinforcing the need for rigorous disinfection protocols, particularly in high-risk areas. Given the lack of standardized cleaning guidelines and the degree of contamination observed, we recommend emphasizing the lack of hygiene in the use of reusable tourniquets and implementing changes in the sectors responsible for cleaning equipment, staff education, and the type of tourniquets used.

#### Implications for Practice

Applying these findings to nursing practice can help to reduce infections and improve healthcare quality. Training nursing staff in best practices for handling and disinfecting reusable equipment is essential to ensure high standards in patient care. The results also emphasize the importance of fostering a culture of safety, with nurses playing a critical role in following best practices and identifying nosocomial infection risks.

## Figures and Tables

**Table 1:** Comparison of the number of colony-forming units per cm^2^ of tourniquet fabric and per plastic part on a Columbia agar plate with 5% sheep blood. The value of 667 was borderline, with an uncountable number of CFUs on microbiological media.

	Type of Data		CFU/cm^2^ Fabric			CFU Plastic		
		Length of Use	Indefinite Time	14 Days	28 Days	Indefinite Time	14 Days	28 Days
	Type of Room							
Emergency department	pediatric room		667	667	256	23	16	8
	pediatric observation		362	667	358	4	10	14
	adult observation		667	667	667	300	23	73
	adult observation		613	667	667	13	20	42
	consultation area		9	327	667	2	3	16
	resuscitation area		667	551	667	61	8	21
**mean**:			**498**	**591**	**547**	**67**	**13**	**29**
Operating theatre	neurologic		7	20	7	1	0	3
	neurologic		36	16	220	3	0	0
	orthopedic		156	18	102	1	1	0
	orthopedic		124	280	29	2	2	2
	orthopedic		120	198	38	16	5	12
	gynecology		4	4	51	0	0	1
	pediatric		244	22	667	0	0	2
	pediatric		13	13	213	0	0	18
	general surgery		13	36	196	0	8	8
	general surgery		53	4	138	0	1	9
**mean**:			**77**	**61**	**166**	**2**	**2**	**6**

**Table 2:** Comparison of the number of colony-forming units per cm^2^ of tourniquet fabric on the MacConkey and King B substrates. The value of 667 was borderline, with an uncountable number of CFUs on microbiological media.

	Type of Data		MacConkey CFU/cm^2^			King B CFU/cm^2^		
		Length of Use	Indefinite Time	14 Days	28 Days	Indefinite Time	14 Days	28 Days
	Type of Room							
Emergency department	pediatric room		709	453	227	667	667	118
	pediatric observation		191	911	487	667	667	231
	adult observation		667	667	667	667	667	373
	adult observation		558	667	667	416	667	649
	consultation area		9	140	667	4	238	667
	resuscitation area		667	280	667	667	607	667
**average CFU/cm^2^:**			**467**	**520**	**564**	**515**	**586**	**451**
Operating theatre	neurologic		0	27	0	11	22	0
	neurologic		13	4	0	33	11	427
	orthopedic		129	27	0	31	4	9
	orthopedic		144	187	0	44	236	0
	orthopedic		56	167	0	64	160	7
	gynecology		0	2	0	0	18	13
	pediatric		33	24	0	131	18	667
	pediatric		7	11	11	18	13	29
	general surgery		2	16	0	7	2	40
	general surgery		20	2	0	27	2	16
**average CFU/cm^2^:**			**40**	**46**	**1**	**37**	**49**	**121**

**Table 3:** The presence of blood stains on the tourniquets depending on whether the blood stains were present on the fabric or plastic part of the tourniquets.

	Emergency Department		Operating Theatre	
	Fabric	Plastic	Fabric	Plastic
**Indefinite time**	2 of 6 tourniquets	1 of 6 tourniquets	3 of 10 tourniquets	0 of 10 tourniquets
**14 days**	3 of 6 tourniquets	2 of 6 tourniquets	2 of 10 tourniquets	2 of 10 tourniquets
**28 days**	2 of 6 tourniquets	1 of 6 tourniquets	2 of 10 tourniquets	1 of 10 tourniquets

## Data Availability

The original contributions presented in the study are included in the article, further inquiries can be directed to the corresponding authors.
